# Symptomatic unilateral pleural effusion: A rare presentation of ovarian hyperstimulation syndrome

**DOI:** 10.4103/0974-1208.63125

**Published:** 2010

**Authors:** Korula George, TK Aleyamma, MS Kamath, A Chandy, Ann M Mangalaraj, K Muthukumar, V Londhe

**Affiliations:** Department of Reproductive Medicine Unit, Christian Medical College, Vellore - 632 004, India

**Keywords:** *In-vitro* fertilization, ovarian hyperstimulation syndrome, pleural effusion

## Abstract

Isolated pleural effusion is a rare presentation of ovarian hyperstimulation syndrome. The pathogenesis of this disorder has not been fully elucidated. It supports the role of systemic factors rather than transudation of fluid from the surface of enlarged ovaries. This article describes a rare case of isolated pleural effusion following controlled ovarian hyperstimulation during an *in-vitro* fertilization cycle.

## CASE REPORT

A 24-year-old woman, married for 6 years, presented as a case of primary infertility for evaluation and treatment. On evaluation, she was diagnosed to have anovulatory dysfunction (polycystic ovarian syndrome) and her husband's semen analysis showed mild abnormality (mild asthenozoospermia). Laparoscopy done earlier had been reported as normal with bilateral tubal patency. In view of the treatment history of multiple ovulation induction with clomiphene, letrozole, and recombinant gonadotrophins, ovulation induction with gonadotrophins (human menopausal gonadotrophin, HMG) and intrauterine insemination (IUI) was planned.

In spite of ovulation induction being induced with low-dose gonadotrophins (HMG) in view of her high-risk status, she had a hyper response. After appropriate counseling regarding the available treatment options, the couple agreed on conversion to *in-vitro* fertilization (IVF).

Ovarian stimulation was continued with an antagonist being added to prevent premature LH surge. As her serum estradiol was >4000 pg/ml when ultrasound evidence of more than three follicles >18 mm was found, coasting was carried out.

A day later, she was given 5000 IU hCG and oocyte retrieval done 35 hours later. Postprocedure, 20% of albumin (100 ml) intravenously was administered slowly over 4 hours.

Patient was closely monitored daily as an outpatient and advised increased fluid intake with strict maintenance of intake and output.

Two days after oocyte retrieval, she presented with breathlessness and on examination was found to have tachypnea and tachycardia with decreased air entry on the right infrascapular region. On abdominal examination, no distension or free fluid could be documented. Abdominal ultrasound showed enlarged ovaries with no free fluid. She was admitted, IV fluids were started, and investigations ordered. Her renal function including serum electrolytes was normal; PCV was 40.2%, with a normal coagulation profile. Next day, she complained of breathlessness, cough, and right-sided chest pain, pleuritic in nature. While the ECG was normal, an X-ray showed evidence of right-sided pleural effusion.

She was transferred to the intensive care unit and thoracocentesis was carried out. 900 ml of pleural fluid was drained and fluid sent for analysis. Cytologic results, culture and staining for bacteria and fungi were negative. She also received low molecular weight heparin (Inj Fragmin 2500 IU subcutaneous) prophylactically, intravenous 20% albumin daily together with antibiotics and oxygen by ventury mask. With conservative management, she improved and maintained oxygen saturation at room temperature. Because of the severe ovarian hyperstimulation syndrome (OHSS), embryo transfer was not done and all 10 blastocysts were frozen on day 5 by vitrification. Repeat chest X-ray after 8 days showed minimal effusion. Patient recovered completely and was discharged.

A year later, three blastocysts were thawed and transferred. Beta hCG done on 12^th^ day after transfer was positive (585 miu/ml). Transvaginal ultrasound done 2 weeks later showed a single viable pregnancy. Estrogen and progesterone support was continued till 12 weeks of gestation. Repeat scan at 14 weeks showed single viable ongoing pregnancy.

## DISCUSSION

OHSS is a serious complication of IVF treatment and can present with varying degrees of severity. The milder forms are associated with a feeling of being unwell, abdominal discomfort, nausea, and vomiting. Ovarian enlargement with tenderness is generally evident. The moderate form includes ultrasonic evidence of ascites (grade 3), in addition to other previously mentioned symptoms. A more severe form is associated with clinically apparent ascites with or without pleural effusion and dyspnea (grade 4) and sometimes presents as a life-threatening situation (grade 5) characterized by additional changes in blood volume, hemoconcentration, coagulation abnormalities, and reduced renal perfusion and function.[1]

Pleural effusion in association with OHSS is indeed a rare complication of ovulation induction. A Canadian study where 771 patients were treated with menotrophins revealed that severe OHSS occurred in 22 patients (3%), pleural effusions occurred in 5 (0.65%), and only 1 required a thoracocentesis (0.12%).[2]

As reported in the study by Gregory *et al*., isolated pleural effusions develop predominantly on the right side.[3] Findings in our case of isolated pleural effusion on the right side are in agreement with the previous case reports.

The exact pathogenesis of OHSS remains poorly understood. This syndrome could be precipitated either by vasoactive peptides or cytokines that have been released into the peritoneal cavity by the ovary or has gained access to the systemic circulation from the corpus luteum and/or serosal vessels.[4] Specifically, interleukin-6 (IL-6) has been shown to be markedly elevated in the follicular fluid, ascitic fluid, pleural fluid, as well as in the serum of patients with severe OHSS.[4,5]

The pathophysiology of massive hydrothorax concurrently with minimal ascitis has been described previously.[5] It has been proposed that the diaphragmatic lymphatics are a route for the transfer of ascites into the pleural space in cases of cirrhosis and Meigs syndrome. Others suggest that the ascitic fluid passes through diaphragmatic anatomical defects entering the pleural space. Multiple macroscopic defects covered with only a thin membrane have been directly observed in the tendinous portion of the diaphragm. Reports have also linked a higher rate of such defects among women. Small unilateral or bilateral pleural effusions can be observed in many patients with moderate and severe forms of OHSS without pulmonary compromise. It would be rather unlikely that a systemic peptide will target its vascular permeability effects on the pleural space unilaterally, without affecting any other serosal membrane. This case, as with the previous reports,[6,7] is interesting because it seems that in the absence of any other pathology, capillary permeability in a single lung has increased.

Twenty-one oocytes were retrieved with a serum estradiol level of >4000 pcg/ml at the time of oocyte pick up. In high-risk cases who are prone for OHSS development, preventive measures such as coasting, GnRh agonist trigger, intravenous albumin, progesterone as luteal support, and recently use of dopaminergic agonists have been advocated. Use of dopaminergic agonists, which acts by reducing vascular endothelial growth factor (VEGF) mediated pathologic increase in permeability, appears promising though larger studies are required to confirm its efficacy.[8]

Importantly in these high-risk cases, it would be a good strategy to culture the embryos till day 5. The crucial time gained would help us identify women who are developing moderate to severe OHSS, and in these cases blastocyst freezing can be offered. An efficient vitrification program would help optimize cycle outcome in such a scenario.

Despite close monitoring during ovarian stimulation, complications like OHSS do occur, although most often in the milder form. This is an iatrogenic complication whose pathophysiology is still not clear and carries a significant morbidity and mortality. This case report was published to highlight a rare clinical presentation of OHSS in the form of unilateral pleural effusion and the need for a multidisciplinary approach.
